# Representative survey on idiopathic environmental intolerance attributed to electromagnetic fields in Taiwan and comparison with the international literature

**DOI:** 10.1186/s12940-018-0351-8

**Published:** 2018-01-15

**Authors:** Po-Chang Huang, Meng-Ting Cheng, How-Ran Guo

**Affiliations:** 1 Department of Environmental and Occupational Health, College of Medicine, National Cheng Kung University, 138 Sheng-Li Road, Tainan, 704 Taiwan; 2 Department of Occupational and Environmental Medicine, National Cheng Kung University Hospital, Tainan, Taiwan

**Keywords:** Electromagnetic field, Electro hypersensitivity, Prevalence, Idiopathic environmental intolerance, Base station, Mobile phone

## Abstract

**Background:**

Electromagnetic hypersensitivity refers to health effects attributed to electromagnetic fields (EMF) exposure and has been formally named “idiopathic environmental intolerance attributed to electromagnetic fields” (IEI-EMF) by the World Health Organization. Because of the growing use of cell phones, IEI-EMF has become a global public health concern. A survey in 2007 in Taiwan showed that the prevalence rate of IEI-EMF was 13.3%, which is higher than rates in studies conducted previously. The survey also found that the rate was higher in women.

**Methods:**

To evaluate whether the prevalence rate of IEI-EMF is increasing and to verify the higher risk in women, we conducted a nationwide questionnaire survey using the same methods as the 2007 survey to assess the change in the prevalence rate of IEI-EMF in Taiwan. We also conducted a review of the literature and a meta-analysis to evaluate the changes in the prevalence rate around the world.

**Results:**

On the basis of the representative sample of 3303 participants, we found that the prevalence rate of IEI-EMF in Taiwan declined from 13.3% to 4.6% over a period of 5 years. The literature review also found the prevalence rates in other countries to be decreasing, instead of increasing as predicted previously. The meta-analysis of the data from the literature showed that women are more likely to have IEI-EMF than men, with an odds ratio of 1.19 (95% confidence interval: 1.01—1.40).

**Conclusions:**

We found the prevalence rate of IEI-EMF has been declining, instead of increasing as predicted previously. Women are more likely to report having IEI-EMF than men. Further studies to explore the causes leading to the declines may help the public, scientific community, and government deal with idiopathic intolerance to other environmental exposures.

## Background

With the extensive usage of electrical devices and wireless apparatuses, humans are inevitably exposed to EMF. Certain individuals believe that exposure to EMF contributes to their discomforts, including both physical and psychological symptoms. These symptoms include headache; fatigue; stress; sleep disturbances; skin symptoms like prickling, burning sensations and rashes; pain and ache in muscles; and many other health problems. They are collectively called electromagnetic hypersensitivity (EHS) [1, 2]. These symptoms may seriously affect the lives of EHS sufferers, and some of them try to avoid EMF and even prohibit others from using mobile phones. In severe cases, this condition may lead to unemployment and isolation from the society [3]. After considering alternative etiologies [4], WHO coined the term “idiopathic environmental intolerance attributed to electromagnetic fields” (IEI-EMF) to denote the symptoms attributed to EMF [5]. IEI-EMF remains primarily as a cluster of self-reported sensitive symptoms without clear clinical diagnostic benchmarks or a clear case definition, Neither in vivo nor in vitro studies have been able to establish the mechanism. Neither dosage nor exposure duration of EMF has been shown to be correlated with the reported symptoms [6–11].

As IEI-EMF constitutes a worldwide public health issue, its prevalence rate has been investigated globally. It was reported in 1998 as 3.2% in California [12] and in 2004 as 5% in Switzerland [13]. A survey in Taiwan in 2007 showed that the prevalence rate was 13.3% [14], much higher than those reported in other countries. An increasing trend was also observed in some other countries. In Sweden, the prevalence rate was reported as 1.5% in 1997 [15] and grew to 2.6%–3.2% in 2001 [16, 17]. In Austria, it was reported as 2% in 1994 [18] and reached 3.5% in 2008 [19]. A review of literature in 2006 found an increasing global trend and predicted the rate is likely to reach 50% in 2017 globally [20]. If so, the impact on public health would be enormous. However, the most recent data included by the review were from as early as 2004, and the prediction was based on the assumption that the prevalence would preserve the same increasing trend. The 2007 Taiwanese survey also found a higher prevalence rate in women, but studies on this issue are limited [21–23]. To evaluate whether the prevalence of IEI-EMF is increasing, we conducted a survey in Taiwan using the same methods as those in the 2007 survey [14] and performed a meta-analysis to assess the global time trend in the prevalence rate. We also conducted a review of the literature on the difference in the risk between the two sexes.

## Methods

### Questionnaire survey

To assess the change in the prevalence rate of IEI-EMF in Taiwan, we conducted a nationwide telephone survey using the same method as in the 2007 survey [14]. The 2007 survey was targeted at the households equipped with a telephone. The participants were randomly selected from the phonebook of Chunghwa Telecom, which is the only wire telephone provider in Taiwan, using the Computer-Assisted Telephone Interviewing System (WinCATI 2000, Sawtooth Technologies) [24]. To ensure that the sample was representative, the survey applied a two-stage, geographically stratified systematic sampling scheme. Households were randomly selected from each of the 25 geographical areas of Taiwan according to proportional population size, and a respondent above 18 years of age was enrolled from each household. We divided the administration districts in Taiwan into the northern, middle, and southern regions. All three regions have similar population sizes. The target sample size was set to obtain about the same number of participants with IEI-EMF as in the 2007 survey (170) in each region. As an increase in the prevalence was predicted, we used the upper bound of the 95% confidence interval (95%CI) of the estimated prevalence in the 2007 survey (15.3%) to estimate the target sample size (170 ÷ 15.3% = 1111) and then rounded off the number to 1100.

The interview consisted of questions regarding demographic variables, presence of catastrophic illness, self-reported health conditions, risk perception of various environmental agents, impairment of daily activities, and medical resource utilization. Self-perceived health status was evaluated with a five-point Likert scale. The EMF sources listed include mobile phone base stations, mobile phones, electric towers, and high voltage cables. IEI-EMF was identified by the question “While being near EMF sources such as mobile phone, electrical devices, or computer, do you feel allergic or sensitive?” The survey reached 5643 households, and 1251 individuals completed the interview.

Telephone interviews were conducted at the Survey and Statistics Research Center, Department of Statistics, National Cheng Kung University between December 2012 and March 2013. As in the 2007 survey, outlying islands including Lienchiang and Kinmen Counties of the Fukien Province were not included. We used the same questionnaire as the one used in the 2007 survey [14], and the response rate was 23.6%, which was compatible to the 22.2% response rate in the 2007 survey.

The study protocol was reviewed and approved by a Grant Review Committee of the Environmental Protection Administration of the Taiwanese government, and participation of human subjects did not occur until after informed consent was obtained.

### Literature review and meta-analysis

With the keywords “electro hypersensitivity,” “EHS,” “idiopathic environmental intolerance,” “IEI-EMF,” “prevalence,” “electromagnetic field,” “base station,” “mobile phone,” and “cellular phone,” we conducted a systematic search of literature using databases including PubMed, ISI Web of Knowledge, and Google Scholar to identify human epidemiology studies on the prevalence rate of IEI-EMF. From a same study, only one article was included. A total of 15 qualified articles were identified [12–15, 19, 22, 23, 25–32], and 3 more reports with relevant data were identified from the references cited in those articles [22, 23, 28]; all of them were published before 2014. We extracted all the data available from each study to perform meta-analyses.

### Statistical analysis

To compare with the results of the 2007 survey, we used the population at the end of 2007 in Taiwan as the reference population to adjust for age, sex, and education [33]. Weighted prevalence rates and their confidence intervals were calculated using the SVYTABLE command of the survey package [34] of R (Version 3.3.2) [35]. Odds ratios (ORs) and their confidence interval associated with the predictor variables were obtained by using the command SVYGLM for weighted logistic regressions. We applied the Breslow-Day test to evaluate the homogeneity of the ORs between the current study and the 2007 study.

We also conducted a meta-analysis of the prevalence of IEI-EMF using R’s Metafor package [36]. We evaluated the between-study heterogeneity using the Ι^2^ statistic. Considering the diversity of the population in different countries and differences in the investigation methods, we adopted the random effect model to estimate the effect size. All statistical tests were performed at a two-tailed significant level of 0.05.

## Results

Among the 3303 telephone-interviewed participants, 155 reported IEI-EMF, yielding a prevalence rate of 4.7% (Table 1). Females comprised the majority (62.6%) of the IEI-EMF sufferers. The IEI-EMF and non-IEI-EMF groups had similar distributions in all the variables studied, except that IEI-EMF sufferers were more likely to report impairment in daily life (23.9% vs. 11.4%, *p* = 0.02) (Table 1).

**Table 1:** Demographic data and other characteristics of participants with and without idiopathic environmental intolerance attributed to electromagnetic fields (IEI-EMF)

Variable	IEI-EMF (*n* = 155) N (%)	Non-IEI-EMF (*n* = 3148) N (%)	Survey population (*n* = 3303) N (%)	Weighted % (95% confidence interval)	
				IEI-EMF	Survey population
Sex					
Female	97 (62.6)	1832 (58.2)	1929 (58.4)	58.8 (50.6, 66.6)	49.6 (47.8, 51.3)
Male	58 (37.4)	1316 (41.8)	1374 (41.6)	41.2 (33.4, 49.4)	50.4 (48.7, 52.2)
Age (year)	47.8 ± 13.63	48.56 ± 14.96	48.53 ± 14.90		
18–34	29 (18.7)	589 (18.7)	618 (18.7)	40.5 (32.8, 48.8)	39.6 (38.0, 41.3)
35–49	49 (31.6)	945 (30.0)	994 (30.1)	29.4 (22.5, 37.4)	28.7 (27.1, 30.2)
50–64	58 (37.4)	1151 (36.6)	1209 (36.6)	19.6 (13.8, 27.0)	19.6 (18.3, 21.0)
≥ 65	19 (12.3)	463 (14.7)	482 (14.6)	10.5 (6.3, 16.7)	12.1 (11.0, 13.3)
Perceived Health Status					
Excellent	7 (4.5)	165 (5.2)	172 (5.2)	3.5 (1.3, 8.1)	5.0 (4.3, 5.8)
Good	33 (21.3)	848 (26.9)	881 (26.7)	23.1 (16.9, 30.8)	23.8 (22.3, 25.3)
Fair	94 (60.6)	1837 (58.4)	1931 (58.5)	57.0 (48.8, 64.9)	60.7 (59.0, 62.4)
Poor	17 (11.0)	261 (8.3)	278 (8.4)	7.9 (4.3, 13.7)	9.0 (8.1, 10.0)
Very poor	4 (2.6)	37 (1.2)	41 (1.2)	8.5 (4.8, 14.4)	1.6 (2.0, 20.9)
Impairment in Daily Activities	37 (23.9)*	513 (11.4)	395 (12)	24.6 (18.2, 32.4)	12.6 (11.5, 13.8)
Education Level					
Middle school and below	38 (24.5)	785 (25.2)	823 (25.2)	47.7 (39.6, 55.9)	40.2 (38.5, 41.9)
High school	41 (26.5)	953 (30.6)	994 (30.4)	29.3 (22.4, 37.3)	38.3 (36.6, 39.9)
College and above	76 (49.0)	1375 (44.2)	1451 (44.4)	23.0 (16.7, 30.6)	21.5 (20.2, 23.0)
Employment Status					
Employed	139 (89.67)	2817 (89.49)	2956 (89.49)	85.3 (78.5, 90.3)	88.1 (87.0, 89.2)
Out of work/not working	10 (6.45)	152 (4.45)	162 (4.90)	7.0 (3.7, 12.5)	6.0 (5.3, 6.9)
Unable to work	6 (3.87)	179 (5.24)	185 (5.60)	7.7 (4.2, 13.4)	5.9 (5.1, 6.7)

*p < 0.05

To compare the results of our survey with those of the 2007 survey, we adjusted the IEI-EMF prevalence rate for sex, education level, and age according to the demographic data in 2007 reported by the government.

In the current study, the weighted prevalence rate was 4.6% (95%CI: 4.0%—5.4%) (Table 1). In comparison with the general population, higher proportions of IEI-EMF sufferers had perceived their health status as “very poor” (8.5% vs. 1.6%, *p* < 0.001) and reported impairment in daily activities (24.6% vs. 12.6%, *p* < 0.001).

Logistic regressions showed that after adjusting for other factors, males had a lower prevalence rate of IEI-EMF, with an adjusted odds ratio (OR) of 0.81, but the difference was not statistically significant (95%CI: 0.58—1.13) (Table 2). A decreasing trend in the prevalence of IEI-EMF (*p* = 0.05) was found as age increased. IEI-EMF was associated with a higher risk of reporting impairment in daily activities (adjusted OR =2.63, 95%CI: 1.69—4.00) (Table 2).

**Table 2:** Odds ratios and 95% confidence intervals from univariate and multi-variate logistic regressions

	Current Survey		
Variable	Univariate analysis	Multivariate analysis	Weighted multivariate analysis
Sex			
Female	1	1	1
Male	0.8 (0.6, 1.2)	0.8 (0.6, 1.1)	0.7 (0.4, 1.1)
Age (year)			
18–34	1	1	1
35–49	1.0 (0.7, 1.7)	1.1 (0.7, 1.9)	0.9 (0.4, 2.0)
50–64	1.0 (0.7, 1.6)	1.1 (0.7, 1.8)	0.8 (0.3, 2.0)
≥ 65	0.8 (0.5, 1.5)	0.8 (0.4, 1.6)	0.6 (0.2, 1.9)
Perceived Health Status			
Excellent	1	1	1
Good	0.9 (0.4, 2.3)	0.9 (0.4, 2.1)	1.3 (0.5, 3.9)
Fair	1.2 (0.6, 2.9)	1.1 (0.5, 2.7)	1.3 (0.5, 3.2)
Poor	1.5 (0.6, 4.0)	1.0 (0.4, 2.8)	0.9 (0.3, 2.6)
Very poor	2.5 (0.6, 8.9)	1.6 (0.4, 5.8)	5.4 (1.4, 20.6)*
Impairment in Daily Activities			
No	1	1	1
Yes	2.4 (1.6, 3.6)*	2.6 (1.7, 4.0)*	2.2 (1.2, 3.8)*
Education Level			
Middle school and below	1	1	1
High school	0.9 (0.6, 1.4)	0.9 (0.6, 1.5)	0.6 (0.3, 1.4)
College and above	1.1 (0.8, 1.7)	1.2 (0.8, 1.9)	0.9 (0.4, 2.0)
Employment Status			
Employed	1	1	1
Out of work/not working	1.3 (0.6, 2.5)	1.4 (0.7, 2.6)	1.0 (0.4, 2.8)
Unable to work	0.7 (0.3, 1.4)	0.6 (0.2, 1.3)	0.9 (0.3, 2.5)

*p < 0.05

Compared to the results of the 2007 survey (Table 3), when the same weights were applied according to age, sex, and education level, our survey did not find a lower risk of IEI-EMF in participants ≥65 years of age. Likewise, we did not find a higher risk of being unable to work in participants with IEI-EMF. However, we found a higher risk of reporting impairment in daily activities (adjusted OR = 2.17, 95%CI: 1.24—3.78) that had not been found significant in the 2007 survey. When we applied tests for homogenicity to determine differences in the results between our survey and the 2007 survey, we found the ORs associated with sex were different between the two surveys (*p* = 0.03 for test for homogennecity) (Table 3). ORs associated with other factors such as age, percevived health status, impariment in daily activities, education level, and employment status were similar between the two surveys.

**Table 3:** Homogeneity test of 2007 and the current study

	Weighted Multivariate Analysis		
Variable	Current survey	2007 survey	Homogeneity test
Sex			*p* = 0.03
Female	1	1	
Male	0.7 (0.4, 1.1)	1.0 (0.7, 1.4)	
Age (year)			*p* = 0.15
18–34	1	1	
35–49	0.9 (0.4, 2.0)	1.2 (0.8, 1.8)	
50–64	0.8 (0.3, 2.0)	1.1 (0.7, 1.8)	
≥ 65	0.6 (0.2, 1.9)	0.4 (0.1, 1.0)	
Perceived Health Status			*p* = 0.12
Excellent	1	1	
Good	1.3 (0.5, 3.9)	0.9 (0.6, 1.4)	
Fair	1.3 (0.5, 3.2)	0.7 (0.4, 1.2)	
Poor	0.9 (0.3, 2.6)	0.8 (0.4, 1.6)	
Very poor	5.4 (1.4, 20.6)*	4.9 (1.6, 15.2)*	
Impairment in Daily Activities			*p* = 0.42
No	1	1	
Yes	2.2 (1.2, 3.8)*	1.3 (0.8, 2.1)	
Education Level			*p* = 0.37
Middle school and below	1	1	
High school	0.6 (0.3, 1.4)	1.1 (0.6, 1.8)	
College and above	0.9 (0.4, 2.0)	1.1 (0.7, 1.8)	
Employment Status			*p* = 0.28
Employed	1	1	
Out of work/not working	1.0 (0.4, 2.8)	1.6 (0.8, 3.0)	
Unable to work	0.9 (0.3, 2.5)	1.8 (1.1, 3.2)*	

*p < 0.05

The prevalence rate of IEI-EMF in Taiwan has not increased since 2007. Instead, we observed a remarkable decrease from 13.3% to 4.6% after adjusting for sex, age, and education level (*p* < 0.001).

**Fig. 1:**
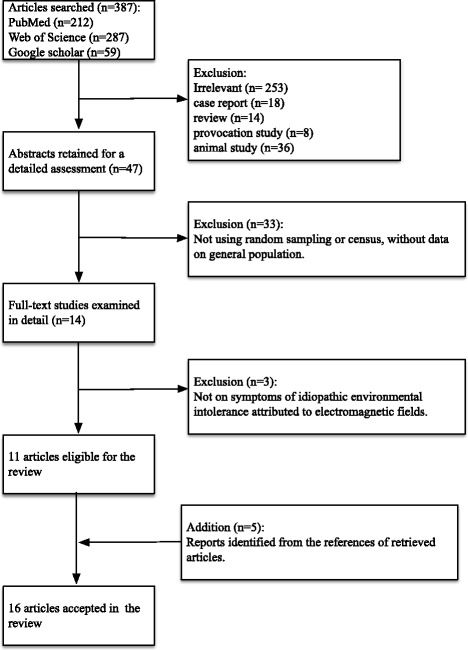
Flow chart of the systematic literature review

**Fig. 2:**
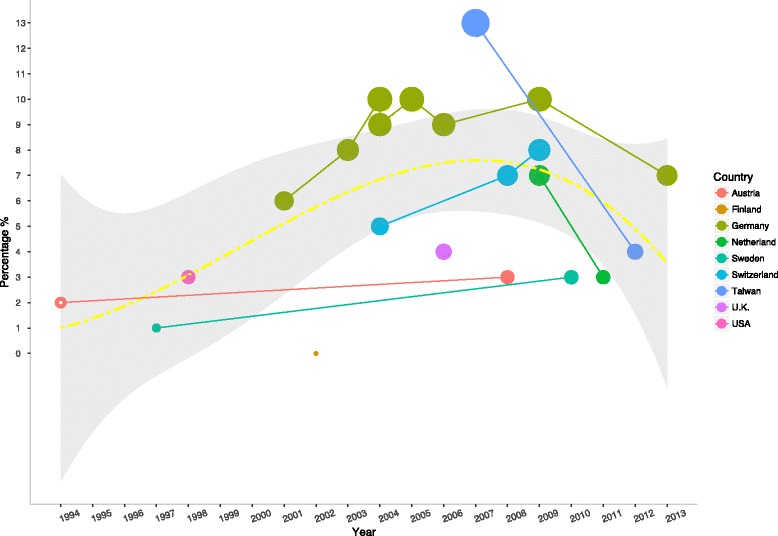
Prevalence rates (%) of idiopathic environmental intolerance attributed to electromagnetic fields around the world. The 1994 data from Austria were reported by Schröttner and Leitgeb (2008), but the actual raw data are unavailable from the references cited in the paper [19]. Therefore, we presented the 2% prevalence rate with a hollow circle

**Fig. 3:**
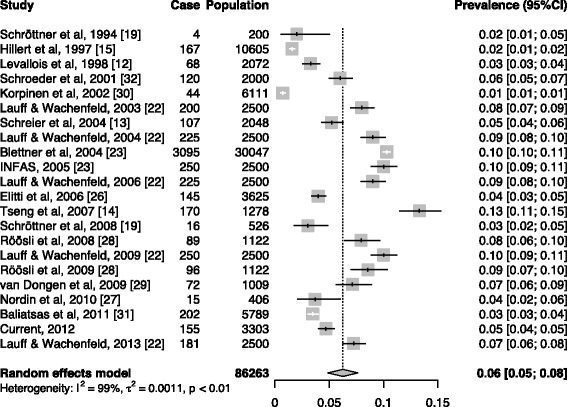
Forest plot of prevalence rates of idiopathic environmental intolerance attributed to electromagnetic fields around the world. I^2^ = 99.4%, *p* < 0.01 for heterogeneous test. The years are the years of investigation

**Fig. 4:**
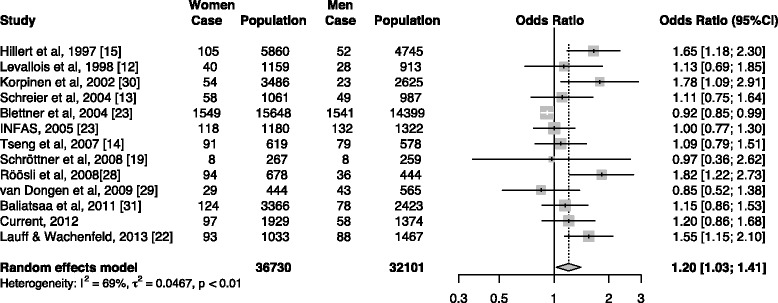
Forest plot of prevalence rates of idiopathic environmental intolerance attributed to electromagnetic fields in men and women. I^2^ = 69.0%, *p* < 0.01 for heterogeneous test. The years are the years of investigation

After a search of the literature on the prevalence of IEI-EMF (Fig. 1), we obtained data on nine countries (Fig. 2) (Table 4). Overall, there was an increasing trend in the prevalence after the first report in 1994, but recently, the prevalence seemed to be in decline. Specifically, prevalence rates in all the three countries with more than one estimate after 2006 in our meta-analysis had declined: from 13.3% in 2007 to 4.6% in 2013 in Taiwan, from 7.0% in 2009 [29] to 3.5% in 2011 [31] in the Netherlands, and from 10.0% in 2009 [22] to 7.0% in 2013 [22] in Germany (Fig. 2). The meta-analysis yielded an estimated overall prevalence rate of 6.0% (95%CI: 5.0%—8.0%), but the results were determined as heterogeneous (*p* < 0.01 for test for homogenicity) (Fig. 3). Our meta-analysis also showed that women were more likely to report IEI-EMF than men, with an summary OR of 1.20 (95%CI: 1.03—1.41) (Fig. 4), but the results among those studies were determined to be heterogeneous (*p* < 0.001 for test for homogenicity). As the test for homogenicity was statistically significant, a random effect model was applied to obtain the pooled prevalence. Our survey results agree with those in most previous studies that females have a slightly higher prevalence of IEI-EMF [13, 15].

**Table 4:** The literatures in the meta-analysis

Reference	Survey year	Method	Country/region	IEI-EMF definition	Case/population
Schröttner et al., 2008 [19]	1994	NA	Austria	NA	4/200
	NA	Telephone survey		Persons were classified as EHS if they reported adverse health effects from EMF sources.	16/526
Hillert et al., 2002 [15]	1997	Mailed questionnaire	Sweden/Stockholm	The respondents were asked to check all factors for which they were hypersensitive or allergic such as electric or magnetic fields.	167/10605
Levallois et al., 2002 [12]	1998	Telephone survey	USA/California	“Allergic or very sensitive to getting near electrical appliances, computers or power lines.”	68/2072
Schroeder et al., 2002 [32]	2001	Telephone survey	Germany	Questionnaire: “Are you worried about the electromagnetic fields emanating from mobile phone systems, cell phones or cordless phones, or are you even affected by these fields in your health?”	120/2000
Korpinen et al., 2009 [30]	2002	Telephone survey	Finland	Self- reported physical symptoms associated with using mobile phones and other electrical devices.	44/6111
Blettner et al., 2009 [25]	2004	Mailed questionnaire	Germany	Whether the participants believe that their health is adversely affected by mobile phone base stations.	3095/30047
Schreier et al., 2006 [13]	2004	Telephone survey	Switzerland	Persons were classified as EHS individuals if they reported adverse health effects from an EMF source at the time of the interview or anytime in the past.	107/2048
Institut für angewandte Sozialwissenschaft GmbH (INFAS), 2006 [23]	2003	Telephone survey	Germany	Questionnaire: “Degree of anxiety and impairment due to electromagnetic fields of mobile radio, referring to different sources of electromagnetic fields, types of impairment.”	200/2500
	2004				225/2500
	2005				250/2502
	2006				225 /2500
Lauff & Wachenfeld, 2014 [22]	2009				250/2500
	2013				181/2500
Tseng et al., 2011 [14]	2007	Telephone survey	Taiwan	“While being near EMF sources such as mobile phone, electrical devices, or computer, will you feel allergic or sensitive?”	170/1278
Röösli et al., 2010 [28]	2008	Web-based questionnaire	Switzerland	“Are you electrohypersensitive?”	96/1122
	2009				86/1122
van Dongen et al., 2014 [29]	2009–2010	Web-based questionn	Netherlands/Amsterdam	“Do you believe you are sensitive to electromagnetic fields?”	72/1009
Nordin et al., 2013 [27]	2010	Mailed questionnaire	Sweden/Västerbotten	The responders self-reported of having been diagnosed as IEI-EMF by a physician.	15/3406
Baliatsas et al., 2014 [31]	2011	Mailed questionnaire	Netherlands	“I am sensitive to mobile phone base stations and devices related to communication systems”; “I am sensitive to electrical devices.”	202/5789
Eltiti et al., 2007 [26]	NA	Mailed questionnaire	United Kingdom	“Are you sensitive to EMFs?”	145/3625
Current	2012–2013	Telephone survey	Taiwan	“While being near EMF sources such as mobile phone, electrical devices, or computer, will you feel allergic or sensitive?”	155/3303

*NA*: not available

## Discussion

Our study found that the global IEI-EMF prevalence rate has declined in recent years, not increased as predicted by Hallberg and Oberfeld [20]. They collected 17 estimates of the prevalence rates from 1985 to 2004 in seven countries and plotted them over time in a normal distribution diagram, which showed that the prevalence rate would be 50% in 2017. The prediction was based on the assumption that the trend over time will not change direction, and it is proven wrong by the data collected in our study, which extended the data collection to 2013 and observed a change in the direction around 2007. It is true though that there was a consistent increasing trend during the study period of Hallberg and Oberfeld. In our study, three countries had more than one estimate after 2007 (Taiwan, Germany, and the Netherland), and all of them showed a declining trend over time. Among the three countries, two were not included by Hallberg and Oberfeld. Nonetheless, even in Germany, which was covered by their study, there was a decline in the prevalence rate from 2009 to 2013.

Scientific evidence bridging EMF and IEI-EMF symptoms has been scarce [1, 11, 37]. Many double-blind provocation studies have been conducted to determine whether people with IEI-EMF can detect the existence of EMF and whether EMF is the cause of their symptoms. However, the results indicated that IEI-EMF sufferers are unable to precisely detect the existence of EMF and that short-term exposure to EMF cannot provoke the IEI-EMF symptoms [6]. Although some believe that the level of environmental EMF exposure is associated with the prevalence of IEI-EMF, a study in European cities found that while the environmental EMF exposure was increasing annually, the EHS prevalence rate was declining [38].

Since we applied the same method as used in the 2007 survey, we could assess the changes in the prevalence of IEI-EMF over time, which has rarely been achieved. Although our study and the survey in 2007 were both nationwide telephone interviews, a strength of our study is that the number of participants was nearly triple the number as in the 2007 survey (3303 vs. 1197). In both surveys, women had a higher prevalence than men, and the respondents reporting IEI-EMF were mainly in the age range of 35 to 64 years, around 70% of all participants. Our study found a higher prevalence of reporting impairment in daily activities by respondents with IEI-EMF (adjusted OR = 2.17, 95%CI: 1.24—3.78), which was not observed in the 2007 survey (adjusted OR = 1.3, 95%CI: 0.8—2.1). However, the proportion of respondents with IEI-EMF who reported being unable to work was 3.9% in our study, much less than the 20% proportion in the 2007 survey. In fact, while the 2007 survey reported that the IEI-EMF group had a higher risk of being unable to work (adjusted OR = 1.8, 95%CI: 1.1—3.2), we did not observe such an increased risk in our survey (adjusted OR = 0.9, 95%CI: 0.3—2.5). The question to inquire participants if their health status contributing to the impairment in daily activities was not classified into more detail items, such as impairment in walking, dressing, writing, reading, and so on. Therefore, the severity of impairment in daily activities cannot be mirrored to the work ability. In addition, although the risk of IEI-EMF sufferers in our study who reported their health status as very poor (adjusted OR = 5.4, 95%CI: 1.4—20.6) was higher than that in the 2007 survey (adjusted OR = 4.9, 95%CI: 1.6—15.2), the differences between the two surveys did not reach statistical significance.

It is possible that very serious IEI-EMF sufferers could not be contacted by our telephone interviews, and consequently, the prevalence rate could be underestimated. However, this should have occured in both surveys and is unlikley to account for the large difference in the prevalence rates. The phonebook of Chunghwa Telecom does not include mobile phones, and therefore our survey might over-estimate the prevalence rate because sufferers of IEI-EMF are less likely to use mobile phones and thus more likely to subscribe landline phones due to the fear of EMF. Since the 2007 survey also used the phonebook and we observed a decrease instead of an increase, our conclusion of a decreasing trend should still hold even if the sample was biased.

Media reports affecting public awareness may partly explain the trend. The media reports on IEI-EMF have focused on precaution and could influence reader’s perception, even in lack of scientific evidence [39–41]. When the media pays less attention to IEI-EMF, the number people attributing their discomfort to EMF may decrease. For example, a study in the Netherlands found that the number of newspaper articles decreased from 87 in the first year (March 2008 to March 2009) to 68 in the second year (March 2009 to March 2010), and a decline in the prevalence of IEI-EMF from 7.0% in 2009 [29] to 3.5% in 2011 [31] was observed (Fig. 2). An alternative explanation for the declining trend is the effects of efforts in managing the public fear of EMF. Beginning with the early reports of IEI-EMF symptoms observed in the 1930s, public health workers have made efforts to verify the causality of EMF exposure to IEI-EMF and to alleviate the tense conflict among the government, general public, scientific community, and industries by setting EMF exposure guidelines and monitoring envionmental EMF [42]. The WHO has compiled many fact sheets, and therefore public knowledge of environmental EMF exposure might have been altered by various efforts in the world.

The declining IEI-EMF prevalence trend might also be attributed to the public’s concern having been turned to other environmental issues such as particulates in the air. Furthermore, it is also possible that humans may develop tolerance of EMF after a period of exposure. A double blind crossover study that investigated the potential effects of mobile phone-like RF-EMF on pain threshold perception in response to thermal stumli observed a reduced desensitization effect between repeated stimulations [43].

Our study showed that women had a higher prevalence of IEI-EMF and constituted 63.6% of the sufferers, and this is consistent with findings in previous studies. For example, Schreier et al. [13] conducted a questionnaire survey in Switzerland and found the proportion to be 54.5%, and Röösli et al. [28] conducted another survey in Switzerland and found the proportion to be 72.3%. In addition, women were found to have a lower perception threshold than men in detecting a 50-Hz electric current [44]. Our meta-analysis also showed that women are more likely to report IEI-EMF than men. Some reserachers believe that women are emotionally more sensitive than men and thus are likely to misattribute their idiopathic symptoms to the exposure of EMF [29, 45]. Women supposedly stay indoors for longer than men because of the nature of their work. Stratifed analysis of job contents between men and women could probably test the hypothesis of static daily activity being correlated with IEI-EMF for further study. An alternative explanation of this finding might be the masculine gender role discourages the expression of pain.

## Conclusions

We found that the prevalence of IEI-EMF has declined remarkably in Taiwan and that the prevalence also seems to be declining globally. Women are more likely to report having IEI-EMF than men. Further studies of the reasons why the prevalence declines may help the public, scientific community, and government deal with idiopathic intorance to other environmental exposures.

## Abbreviations

EHS: Electromagnetic hypersensitivity
EMF: Electromagnetic fields
IEI-EMF: Idiopathic environmental intolerance attributed to electromagnetic fields
OR: Odds ratio
RF-EMF: Radio-frequency electromagnetic field

## References

[1] Rubin GJ, Das Munshi J, Wessely S (2006). A systematic review of treatments for electromagnetic hypersensitivity. Psychother Psychosom.

[2] Baliatsas C, Van Kamp I, Lebret E, Rubin GJ (2012). Idiopathic environmental intolerance attributed to electromagnetic fields (IEI-EMF): a systematic review of identifying criteria. BMC Public Health.

[3] Johansson O (2015). Electrohypersensitivity: a functional impairment due to an inaccessible environment. Rev Environ Health.

[4] Genuis SJ, Lipp CT (2012). Electromagnetic hypersensitivity: fact or fiction?. Sci Total Environ.

[5] Hillert L (2004). Report on characterization, diagnosis and treatment. WHO workshop on electrical hypersensitivity.

[6] Wallace D, Eltiti S, Ridgewel A, Garner K, Russo R, Sepulveda F (2010). Do TETRA (airwave) Base Station signals have a short-term impact on health and well-being? A randomized double-blind provocation study. Environ Health Perspect.

[7] Hillert L, Akerstedt T, Lowden A, Wiholm C, Kuster N, Ebert S, Boutry C, Moffat SD, Berg M, Arnetz BB (2008). The effects of 884 MHz GSM wireless communication signals on headache and other symptoms: an experimental provocation study. Bioelectromagnetics.

[8] Oftedal G, Straume A, Johnsson A, Stovner LJ (2007). Mobile phone headache: a double blind, sham-controlled provocation study. Cephalalgia.

[9] Rubin GJ, Hahn G, Everitt BS, Cleare AJ, Wessely S (2006). Are some people sensitive to mobile phone signals? Within participants double blind randomised provocation study. Br Med J.

[10] Regel SJ, Negovetic S, Roeoesli M, Berdinas V, Schuderer J, Huss A, Lott U, Kuster N, Achermann P (2006). UMTS base station-like exposure, well-being, and cognitive performance. Environ Health Perspect.

[11] Rubin GJ, Nieto-Hernandez R, Wessely S (2010). Idiopathic environmental intolerance attributed to electromagnetic fields (formerly ‘electromagnetic hypersensitivity’): an updated systematic review of provocation studies. Bioelectromagnetics.

[12] Levallois P, Neutra R, Lee G, Hristova L (2002). Study of self-reported hypersensitivity to electromagnetic fields in California. Environ Health Perspect.

[13] Schreier N, Huss A, Röösli M (2006). The prevalence of symptoms attributed to electromagnetic field exposure: a cross-sectional representative survey in Switzerland. Soz Praventivmed.

[14] Tseng M-C, Lin Y-P, Cheng T-J (2011). Prevalence and psychiatric comorbidity of self-reported electromagnetic field sensitivity in Taiwan: a population-based study. J Formos Med Assoc.

[15] Hillert L, Berglind N, Arnetz BB, Bellander T (2002). Prevalence of self-reported hypersensitivity to electric or magnetic fields in a population-based questionnaire survey. Scand J Work Environ Health.

[16] Johansson O (2006). Electrohypersensitivity: state-of-the-art of a functional impairment. Electromagn Biol Med..

[17] The National Board of Health and Welfare (Swedish) (2001). Miljöhälsorapport.

[18] Leitgeb N (1994). Electromagnetic hypersensitivity. Quantitative assessment of an ill-defined problem. COST 244 proc.

[19] Schröttner J, Leitgeb N. Sensitivity to electricity - temporal changes in Austria. BMC Public Health. 2008. 10.1186/1471-2458-8-310. 10.1186/1471-2458-8-310 PMC2562386 18789137

[20] Hallberg O, Oberfeld G (2006). Letter to the editor: will we all become electrosensitive?. Electromagn Biol Med.

[21] van Wijk CM, Kolk AM (1997). Sex differences in physical symptoms: the contribution of symptom perception theory. Soc Sci Med.

[22] Lauff H, Wachenfeld A. Abschlussbericht: Differenzierte Betrachtung der Nutzung und der Wahrnehmung des Mobilfunks. Bundesamt für Strahlenschutz (BfS). 2014;BfS-RESFOR-88/14. Retrieved from http://doris.bfs.de/jspui/bitstream/urn:nbn:de:0221-2014022811170/3/BfS_2014_FM8854.pdf. Accessed Jan 2016.

[23] Institut far Angewandte Sozialwissenschaft GmbH (INFAS) (2006). Ermittlung der Beforchtungen und Angste der breiten Offentlichkeit hinsichtlich möglicher Gefahren der hochfrequen-ten elektromagnetischen Felder des Mobilfunks: Abschlussbericht ober die Befragung im Jahr.

[24] Hung Y-T, Huang Y-C (2000). Telephone sampling: random digit dialing in Taiwan. Elect Stud.

[25] Blettner M, Schlehofer B, Breckenkamp J, Kowall B, Schmiedel S, Reis U (2009). Mobile phone base stations and adverse health effects: phase 1 of a population-based, cross-sectional study in Germany. Occup Environ Med.

[26] Eltiti S, Wallace D, Zougkou K, Russo R, Joseph S, Rasor P (2007). Development and evaluation of the electromagnetic hypersensitivity questionnaire. Bioelectromagnetics.

[27] Nordin S, Palmquist E, Claeson AS, Stenberg B. The environmental hypersensitivity symptom inventory: metric properities and normative data from apopulation-based study. Arch Public Health. 2013. 10.1186/0778-7367-71-18. 10.1186/0778-7367-71-18 PMC3716632 23837629

[28] Röösli M, Mohler E, Frei P (2010). Sense and sensibility in the context of radiofrequency electromagnetic field exposure. Comptes Rendus Physique.

[29] van Dongen D, Smid T, Timmermans DRM (2014). Symptom attribution and risk perception in individuals with idiopathic environmental intolerance to electromagnetic fields and in the general population. Perspect Public Health.

[30] Korpinen LH, Pääkkönen RJ (2009). Self-report of physical symptoms associated with using mobile phones and other electrical devices. Bioelectromagnetics.

[31] Baliatsas C, van Kamp I, Hooiveld M, Yzermans J, Lebret E (2014). Comparing non-specific physical symptoms in environmentally sensitive patients: prevalence, duration, functional status and illness behavior. J Psychosom Res.

[32] Schroeder E (2002). Ergebnisse der bundesweiten Telefonumfrage im Auftrag des Bundesamtes für Strahlenschutz. Stakeholder-Perspektiven zur Novellierung der 26 BImSchV [Internet].

[33] Little RJA (1993). Post-stratification: a Modeler's perspective. J Am Stat Assoc.

[34] Lumley T. Analysis of complex survey samples. J Stat Softw. 2004;9:1-19.

[35] Team. RC (2016). R: a language and environment for statistical computing.

[36] Viechtbauer W (2010). Conducting meta-analyses in R with the metafor package. J Stat Softw.

[37] Eltiti S, Wallace D, Russo R, Fox E (2015). Aggregated data from two double-blind base station provocation studies comparing individuals with idiopathic environmental intolerance with attribution to electromagnetic fields and controls. Bioelectromagnetics.

[38] Urbinello D, Joseph W, Verloock L, Martens L, Röösli M (2014). Temporal trends of radio-frequency electromagnetic field (RF-EMF) exposure in everyday environments across European cities. Environ Res.

[39] Claassen L, Smid T, Woudenberg F, Timmermans DRM (2012). Media coverage on electromagnetic fields and health: content analysis of Dutch newspaper articles and websites. Health Risk Soc.

[40] Eldridge-Thomas B, Rubin GJ (2013). Idiopathic environmental intolerance attributed to electromagnetic fields: a content analysis of British newspaper reports. PLoS One.

[41] Huiberts A, Hjornevik M, Mykletun A, Skogen JC (2013). Electromagnetic hypersensitivity (EHS) in the media - a qualitative content analysis of Norwegian newspapers. JRSM Short Rep.

[42] International Commission on Non-Ionizing Radiation P (2009). ICNIRP statement on the “guidelines for limiting exposure to time-varying electric, magnetic, and electromagnetic fields (up to 300 GHz)”. Health Phys.

[43] Vecsei Z, Csatho A, Thuroczy G, Hernadi I (2013). Effect of a single 30 min UMTS mobile phone-like exposure on the thermal pain threshold of young healthy volunteers. Bioelectromagnetics.

[44] Leitgeb N, Schrottner J (2003). Electrosensibility and electromagnetic hypersensitivity. Bioelectromagnetics.

[45] Fillingim RB, King CD, Ribeiro-Dasilva MC, Rahim-Williams B, 3rd Riley JL (2009). Sex, gender, and pain: a review of recent clinical and experimental findings. J Pain.

